# Comparative analysis of perioperative outcomes between hybrid system and MiECC: A prospective pilot study

**DOI:** 10.1051/ject/2025001

**Published:** 2025-06-16

**Authors:** Kaan Kırali, Mehmet Aksüt, Özge Altaş, Mustafa Emre Gürcü, Sibel Aydın

**Affiliations:** 1 Department of Cardiovascular Surgery, Koşuyolu High Specialization Education and Research Hospital Cevizli Kavşağı 34865 Kartal Istanbul Türkiye; 2 Department of Perfusion, Koşuyolu High Specialization Education and Research Hospital Cevizli Kavşağı 34865 Kartal Istanbul Türkiye; 3 Department of Anesthesiology, Koşuyolu High Specialization Education and Research Hospital Cevizli Kavşağı 34865 Kartal Istanbul Türkiye

**Keywords:** Hybrid system, Minimally invasive extracorporeal circulation, Cardiopulmonary bypass, Cardiac surgery, Coronary artery bypass grafting, Transfusion rates

## Abstract

*Background*: Minimally invasive extracorporeal circulation (MiECC) has been introduced to mitigate the inflammatory response and reduce blood transfusion needs compared to conventional cardiopulmonary bypass (CPB) perioperatively. A hybrid system (HS) that merges aspects of both traditional CPB and MiECC aims to optimize patient perioperative outcomes. This study focuses on comparing the postoperative transfusion rates, intensive care unit (ICU) course, and biochemical parameters between the HS and MiECC. *Materials and methods*: This prospective, randomized, controlled, single-center study was conducted at Koşuyolu High Specialization Education and Research Hospital, Istanbul from February 2024 to June 2024. Forty patients undergoing isolated coronary artery bypass grafting (CABG) were included, with 20 patients in the HS-group and 20 in the MiECC-group. Data on oxygen delivery management, hemoglobin and platelet values trends, biochemical parameters, the number of red blood cells and platelet units transfused postoperatively, and ICU stay duration were collected. *Results:* The CPB time was not significantly shorter in the HS group compared to the MiECC group (93.35 ± 33.06 min vs. 108.65 ± 30.02 min, *p* = 0.134). Hemoglobin levels did not differ significantly between the groups preoperatively, perioperatively, or postoperatively at 6, 12, and 24 h no difference in red blood cells unit transfusion. Indexed oxygen delivery did not differ significantly between the HS and MiECC groups (311.60 ± 28.29 mL/min/m^2^ vs. 332.25 ± 57.04 mL/min/m^2^, *p* = 0.275). Partial pressure of oxygen was higher in the MiECC group (210.90 ± 49.64 mmHg vs. 177.70 ± 70.41 mmHg, *p* = 0.093), but this difference was also not statistically significant. Biochemical parameters showed notable differences. Postoperative lactate levels were significantly lower in the HS group (2.85 ± 1.20 mmol/L vs. 4.04 ± 1.40 mmol/L, *p* = 0.009). Conversely, Lactate Dehydrogenase levels during and after CPB were, lower in the MiECC group. Postoperative 6th-hour troponin levels were significantly lower in the HS group (3.188 ± 2.684 ng/mL vs. 4.645 ± 3.422 ng/mL, *p* = 0.038). Mechanical ventilation duration, ICU stay, and hospital stay were comparable between the two groups, with no significant differences observed. *Conclusions*: The hybrid system demonstrated comparable results to the MiECC in patients undergoing isolated CABG. No significant differences were observed in CPB time or postoperative blood transfusion requirements. However, the HS group showed favorable biochemical parameters, including significantly lower postoperative lactate levels and troponin levels at 6 h. Indexed oxygen delivery and partial pressure of oxygen were similar between groups, and ICU and hospital stay durations were comparable. These findings suggest that the hybrid system offers outcomes on par with the MiECC approach, with potential benefits in terms of biochemical markers. Further studies with larger sample sizes are needed to validate these results and explore possible advantages in broader clinical settings.

## Introduction

**Figure 1:**
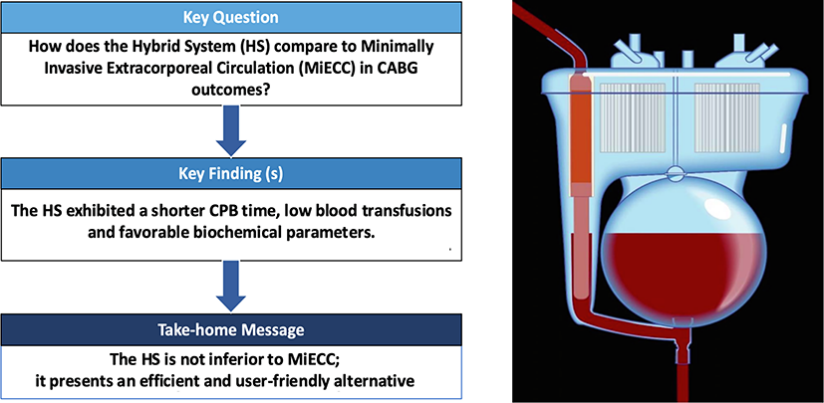
Graphical abstract.

Cardiopulmonary bypass (CPB) has been a cornerstone in cardiac surgery, particularly in procedures such as coronary artery bypass grafting (CABG). Traditional CPB techniques, while effective in maintaining circulation and oxygenation during open heart surgery, are associated with a substantial inflammatory response and a significant need for blood transfusions. This inflammatory response can lead to various postoperative complications, including prolonged recovery times and increased morbidity [1]. To address these issues, the Hybrid System (HS) and Minimally Invasive Extracorporeal Circulation (MiECC) have been developed, offering innovative solutions aimed at reducing these adverse effects. The HS innovative design could play a crucial role in managing systemic inflammatory response syndrome (SIRS), a common complication in patients undergoing CPB. SIRS is characterized by widespread inflammation that can lead to multiple organ dysfunction and increased morbidity [2, 3]. One of the primary triggers of SIRS during CPB is the exposure of blood to foreign surfaces and air, which activates the body’s inflammatory pathways. By incorporating a dual chamber and collapsible soft bag, the HS reduces the blood-air interface and the exposure to foreign materials. MiECC systems integrate several advancements, such as reduced priming volumes, minimized air-blood interface, and optimized blood flow patterns, all contributing to better patient outcomes. Despite these benefits, MiECC is not without limitations, and its application may not be suitable for all patients. In response to these limitations, the HS has been proposed, combining elements of both traditional CPB and MiECC [1, 3]. This hybrid approach aims to leverage the strengths of both systems, optimizing patient outcomes by reducing complications associated with inflammation and transfusions while maintaining the efficacy of traditional CPB (Figure 1). In addition to these mechanical and procedural advancements, biochemical parameters such as lactate, creatinine, troponin, and C-reactive protein levels provide important insights into the physiological impact of CPB and its alternatives [4, 5]. Monitoring these parameters can help in assessing the effectiveness of different CPB techniques in mitigating systemic inflammation and organ dysfunction.

This prospective, randomized, controlled study, first worldwide study, aimed to compare the perioperative outcomes of patients undergoing on-pump isolated CABG using two different extracorporeal circulation systems: HS-group versus MiECC-group.

## Materials and methods

This prospective, randomized, controlled, single-center study was conducted at Koşuyolu High Specialization Education and Research Hospital, Istanbul, Türkiye from February 2024 to June 2024. The study protocol was approved by the Institutional Review Board (IRB) of Koşuyolu High Specialization Education and Research Hospital, and written informed consent was obtained from all participants prior to enrollment. This study was conducted in accordance with the principles outlined in the Declaration of Helsinki. Forty patients scheduled for isolated CABG requiring CPB were enrolled in this study based on the following inclusion (elective surgery, ability to provide informed consent) and exclusion criteria (emergency or urgent surgery, reoperation, known coagulation disorders). Patients meeting these criteria were randomly assigned to either the HS-group (*n* = 20) or the MiECC-group (*n* = 20) using a computer-generated randomization sequence.

### Perfusion techniques

**Figure 2:**
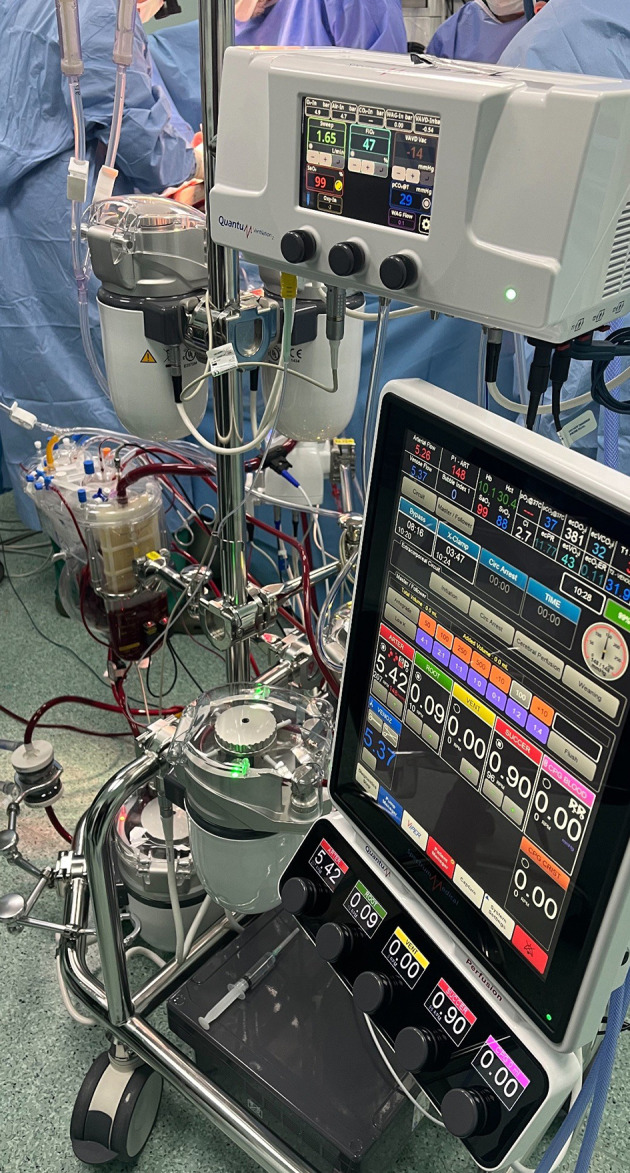
Perioperative use of minimal invasive extracorporeal circulation.

*MiECC group*: The MiECC system is an extra-corporeal technique without a dynamic reservoir, which is a key distinction from the HS. The absence of a reservoir in MiECC means that blood volume control is inherently more restrictive, as it relies on a closed, minimized circuit. This design minimizes the priming volume, reducing blood dilution and limiting fluid exposure. In this context, the MiECC group used Type I models like Extracorporeal Membrane Oxygenation (ECMO) configuration (Figure 2) from MiECTIS classification [6].

**Figure 3:**
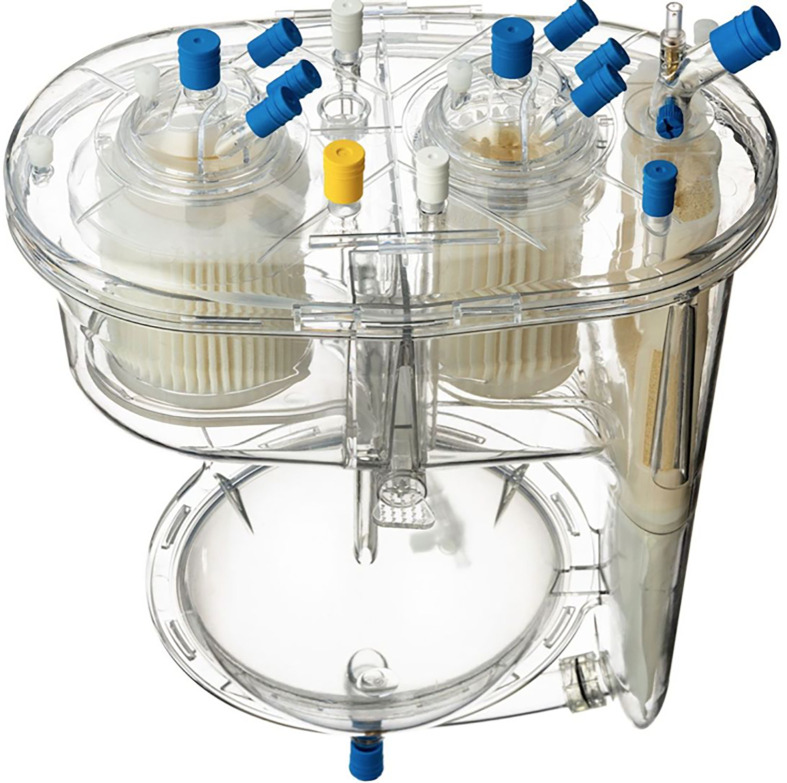
Hybrid system features.

**Figure 4:**
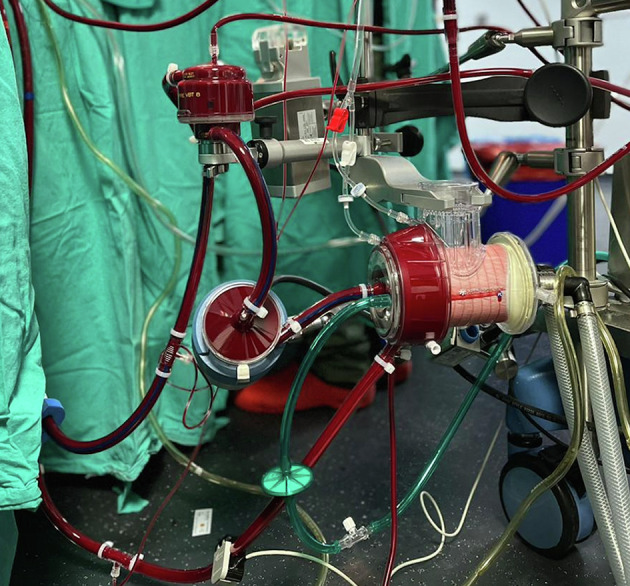
Perioperative use of hybrid system.

*The HS group*: The HS includes a dual-chamber design with a collapsible soft bag that functions as a controlled, adjustable reservoir, which reduces blood-air interface and exposure to foreign materials minimizing systemic inflammatory response (SIRS) risks and reduce gaseous micro-emboly (GE) delivery (Figure 3). This setup allows for better real-time volume adjustments, enhancing the flexibility and adaptability of volume control during surgery. By integrating this reservoir, the HS mitigates the volume management limitations of the MiECC system (Figure 4). The extracorporeal techniques components and features are listed in (Table 1).

**Table 1:** Extracorporeal techniques details.

**Hybrid System Components:** The Hybrid System employed in the study was manufactured by Spectrum Medical and featured advanced components aimed at improving patient outcomes (Figure 1). Key components included:	•*Hybrid Venous Reservoir:* Includes a dual chamber for efficient blood suction management and a collapsible soft bag to minimize blood contact with foreign materials and reduce air exposure (Figure 2).
•*Oxygenator:* Spectrum Medical VT200, providing efficient oxygenation throughout the surgical procedure.	•*Heat Exchanger:* Quantum Standard Heat Exchanger High Flow 3/8, ensuring precise thermal control.
•*Circuit Management:* Filled with 1000 mL crystalloid solution and administered with 300 IU/kg of sodium heparin to achieve an activated clotting time of 480’’ s prior to CPB.	•*Myocardial Protection:* Utilized del Nido solution cardioplegia, administered every 90 minutes via a closed circuit with heat exchanger ensuring myocardial protection throughout the procedure.
•*Perfusion System:* Quantum perfusion system, delivering reliable blood flow with online continuous blood measurement via VIPER (Perfusion Record).	•*Ventilation Module:* Quantum ventilation module, supporting optimal gas blending and management of atmospheric and hypobaric ventilation.
**MiECC Components:** The MiECC system employed a closed type III circuit using the Stöckert S5 heart-lung machine (LivaNova, London, UK) (Figure 3). Key components included:	
•*Venous-Arterial Line:* Diameter 3/8, integrated with a venous bubble-trap (LivaNova, London, UK).	•*Centrifugal Pump:* Revolution Pump (LivaNova, London, UK).
•*Oxygenator:* Polypropylene fiber oxygenator (Ispire 6 F, LivaNova, London, UK) with Biopassive Coating Phisio (LivaNova, London, UK).	•*Bubble Detection System:* Used to remove air from the bubble trap and the circuit (Stöckert, LivaNova).
•*Circuit Management:* Filled with 1000 mL crystalloid solution and administered with 300 IU/kg of sodium heparin to achieve an activated clotting time of 480’’ s prior to CPB.	•*Myocardial Protection:* Utilized del Nido solution cardioplegia, administered every 90 minutes via a closed circuit with heat exchanger ensuring myocardial protection throughout the procedure.
•*Monitoring:* DO_2_ management monitoring was used during CPB, alongside arterial blood gas analysis using alpha-stat management at 37 °C.	

### Surgical technique

All CABG procedures were performed by the same primary surgeon to ensure consistency across cases. After the standard full median sternotomy and systemic heparinization, the circuit was established through arterial and venous cannulation. An aortic cannula (20 or 22 Fr) was inserted into the ascending aorta for systemic arterial blood flow, and an atrial two-stage venous cannula (32/40 Fr) was used to facilitate venous return from the right atrium and vena cava inferior. Cardioplegia was delivered using a 7 Fr needle placed in the ascending aorta.

For myocardial protection, 1250 mL hypothermic (<10 °C) Del Nido cardioplegia solution was administered to achieve and maintain myocardial arrest. Procedures were conducted under mild hypothermia, maintaining the patient’s temperature at approximately 34 °C to enhance myocardial and neuroprotective effects. Conduits, such as the internal mammary artery and/or saphenous vein grafts, were harvested and grafted onto the coronary arteries distal to obstructive lesions, with meticulous attention to the distal anastomosis to ensure optimal graft patency and blood flow. Following grafting, the patient was gradually weaned from CPB. The protamine was administered to reverse heparin effects. Hemodynamic stability was supported with inotropic or vasopressor agents as necessary, and the cannulas were removed carefully. Hemostasis was confirmed, the chest was irrigated, and the sternum was closed in layers. The patient was then transferred to the intensive care unit (ICU) for postoperative monitoring.

### Anesthesia management

Preoperative assessment includes optimizing comorbidities, fasting per ASA guidelines, and premedication with midazolam (1–2 mg IV) as needed. Induction includes standard monitoring (electrocardiography, pulse oximetry, arterial line, central venous access), Bispectral Index (BIS) to monitor anesthesia depth, and Near-Infrared Spectroscopy (NIRS) for cerebral oxygenation. Initial medications include midazolam (0.03–0.05 mg/kg IV), fentanyl (5–10 μg/kg IV), and either propofol (0.5–1 mg/kg IV) or etomidate (0.2–0.3 mg/kg IV) based on patient stability, followed by rocuronium (0.6–1 mg/kg IV) for intubation. Maintenance of anesthesia involves isoflurane or sevoflurane (0.5–1.5 MAC) or TIVA, with continuous infusion of fentanyl (1–5 μg/kg/h) and rocuronium, guided by BIS to maintain optimal depth and NIRS to monitor brain oxygenation. Hemodynamic stability is closely monitored with the transesophageal echocardiography and managed using vasoactive. During CPB, heparin was administered to maintain anticoagulation, and anesthesia was adjusted while closely monitoring acid-base balance. Upon separation from CPB, protamine is administered to reverse heparin, and hemodynamic stability is re-established. For emergence, neuromuscular blockade is reversed, and the patient is transferred to the ICU while sedated and intubated for postoperative monitoring.

### Data collection

Data collected included preoperative demographics (age, gender, left ventricular function), intraoperative parameters such as CPB and aortic cross-clamp time (ACC), and indexed oxygen delivery (DO_2_i), partial arterial oxygen pressure (PaO_2_), and postoperative outcomes including transfusion requirements (red blood cells and platelets), duration of mechanical ventilation, ICU stay duration, and hospital length of stay. Specifically, biochemical parameters such as lactate, creatinine, troponin, C-reactive protein (CRP), lactate dehydrogenase (LDH) and hematologic values (hemoglobulin, platelet) were measured preoperatively, intraoperatively, and at 6, 12, and 24 h postoperatively to assess the physiological impact of the different CPB techniques.

#### Statistical analysis

Continuous variables were presented as mean ± standard deviation (SD) and compared using independent *t*-test or Mann-Whitney *U-*test where appropriate. Categorical variables were presented as frequencies and percentages and were compared using chi-square (χ^2^) test. A *p*-value of less than 0.05 was considered statistically significant.

## Results

**Figure 5:**
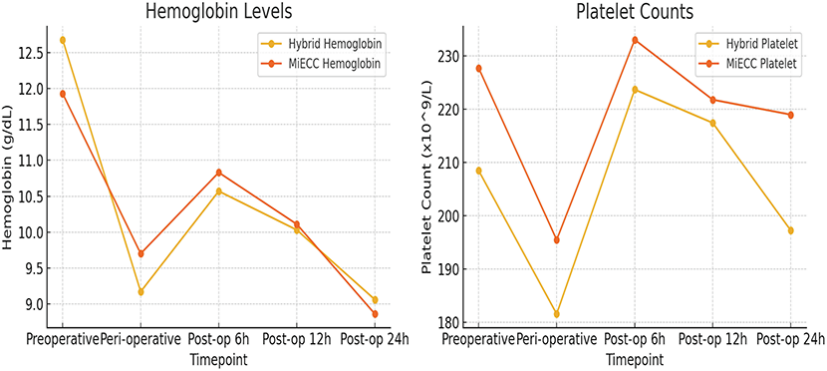
Graphical representation of hemoglobin levels and platelet counts.

The demographic and clinical characteristics, including age, gender distribution, body surface area, and left ventricular ejection fraction, were comparable between the groups with no significant differences (Table 2). The HS-group demonstrated a shorter but nonsignificant CPB and ACC times compared to the MiECC-group. Both groups had an equal number of anastomoses, averaging 3 ± 1 per patient (Table 3). Hemoglobin levels showed no significant differences preoperatively, peri-operatively, and postoperatively at 6, 12, and 24 h between the groups (Figure 5). Platelet counts were also similar between the groups at all measured time points. Red blood cell and platelet transfusion number were similar in both groups (Table 4). Indexed oxygen delivery was in the HS Group (311.60 ± 28.29 mL/min/m^2^) compared to the MiECC Group (332.25 ± 57.04 mL/min/m^2^); the pump flow rate was in the HS group (5.13 ± 0.44 L/min/m^2^) compared to the MiECC Group (4.56 ± 0.25 L/min/m^2^). Conversely, PaO_2_ was higher in the MiECC Group, because HS used the continuous PaO_2_ monitoring and management system (Ventilation/Blood Flow Ratio Auto-initiation mode) from Spectrum Heart Lung Machine to prevent hyperoxia (210.90 ± 49.64 mmHg vs. 177.70 ± 70.41 mmHg, *p* = 0.093). Mean arterial pressure showed no significant difference between the groups (58.55 ± 3.97 mmHg vs. 57.89 ± 4.22 mmHg, *p* = 0.61) (Table 5). Biochemical parameters indicated notable differences between the groups. Lactate levels were statistically lower in the HS Group at the 6th hour after CPB (2.85 ± 1.20 mmol/L vs. 4.04 ± 1.40 mmol/L, *p* = 0.009). Lactate Dehydrogenase levels and trend during and after CPB was lower in the MiECC group than the HS group (Table 6).

**Table 2:** Patient characteristics. Demographic and clinical characteristics of patients in the Hybrid System and MiECC groups.

Characteristic	Hybrid system (*n* = 20)	MiECC (*n* = 20)	*P*-value
Mean age (years)	63.9 ± 6.96	59.2 ± 8.09	0.056
Male sex	18 (90%)	17 (85%)	0.633
Mean body surface area (m^2^)	1.91 ± 0.11	1.94 ± 0.13	0.478
Mean left ventricular ejection fraction (%)	59.2 ± 11.84	58 ± 10.05	0.721

MiECC: minimal invasive extracorporeal circulation.

**Table 3:** Surgical procedures and CPB duration. Distribution of surgical procedures and mean duration of cardiopulmonary bypass in each group.

	Hybrid system (*n* = 20)	MiECC (*n* = 20)	*P* value
Coronary artery bypass grafting	20	20	1.00
Number Anastomoses	3 ± 1	3 ± 1	1.00
CPB time (min.)	93.35 ± 33.06	108.65 ± 30.02	0.134
Cross clamp time (min.)	54.30 ± 25.4	63.7 ± 17.3	0.181

CPB, cardiopulmonary bypass; MiECC, Minimal Invasive Extracorporeal Circulation.

**Table 4:** Changes in laboratory parameters. Comparison of preoperative and postoperative laboratory parameters, including mean hemoglobin levels, mean platelet counts in both groups.

Hemoglobin	Hybrid system (*n* = 20)	MiECC (*n* = 20)	*P* value
Preoperative Hemoglobin levels (g/dL)	12.07 ± 1.96	11.99 ± 1.59	0.895
Peri-operative Hemoglobin levels (g/dL)	8.59 ± 1.38	9.88 ± 1.60	0.10
Post-operative Hemoglobin levels (6 h) (g/dL)	10.26 ± 1.16	10.76 ± 1.38	0.229
Post-operative Hemoglobin levels (12 h) (g/dL)	9.96 ± 0.94	10.03 ± 1.19	0.850
Post-operative Hemoglobin levels (24 h) (g/dL)	9.05 ± 0.84	9.03 ± 1.13	0.938
Platelets	Hybrid system (*n* = 20)	MiECC (*n* = 20)	*P* value
Preoperative platelet counts (×10^9^/L)	209.65 ± 49.67	222.80 ± 75.14	0.518
Peri-operative platelet counts (×10^9^/L)	163.50 ± 58.72	199.90 ± 63.59	0.068
Post-operative platelet counts (6 h) (×10^9^/L)	216.45 ± 75.62	233.10 ± 70.56	0.476
Post-operative platelet counts (12 h) (×10^9^/L)	213.25 ± 65.41	224.10 ± 65.14	0.602
Post-operative platelet counts (24 h) (×10^9^/L)	196.30 ± 61.12	216.00 ± 67.18	0.338

MiECC, Minimal Invasive Extracorporeal circulation;

**Table 5:** Cardiopulmonary bypass parameters comparison.

Cardiopulmonary bypass parameters	Hybrid system (*n* = 20)	MiECC (*n* = 20)	*P* value
Indexed Oxygen Delivery (mL/min/m^2^)	311.60 ± 28.29	332.25 ± 57.04	0.275
Partial Pressure of Oxygen (mmHg)	177.70 ± 70.41	210.90 ± 49.64	0.093
Mean Arterial Pressure (mmHg)	58.55 ± 3.97	57.89 ± 4.22	0.61

MiECC, Minimal Invasive Extracorporeal circulation.

**Table 6:** Pre, intra and postoperative biochemical parameters trend comparison.

	Hybrid system (*n* = 20)	MiECC (*n* = 20)	*P* value
Lactate (before CPB)	1.54 ± 0.54	1.68 ± 0.60	0.324
Lactate (on CPB)	2.00 ± 0.90	2.11 ± 0.85	0.597
Lactate (6 h)	2.85 ± 1.20	4.04 ± 1.40	0.009
Lactate (12 h)	3.01 ± 1.51	3.51 ± 1.76	0.490
Lactate (24 h)	1.96 ± 0.68	2.50 ± 0.93	0.051
Creatinine (before CPB) (mg/dL)	0.91 ± 0.30	0.98 ± 0.87	0.273
Creatinine (on CPB) (mg/dL)	0.75 ± 0.25	0.87 ± 0.80	0.768
Creatinine (6 h) (mg/dL)	0.99 ± 0.34	1.02 ± 0.84	0.636
Creatinine (12 h) (mg/dL)	1.02 ± 0.43	1.09 ± 0.90	0.968
Creatinine (24 h) (mg/dL)	1.05 ± 0.59	0.97 ± 0.75	0.223
Troponin (before CPB) (ng/mL)	0.021 ± 0.031	0.012 ± 0.013	0.356
Troponin (on CPB) (ng/mL)	1.005 ± 2.289	0.917 ± 0.919	0.152
Troponin (6 h) (ng/mL)	3.188 ± 2.684	4.645 ± 3.422	0.038
Troponin (12 h) (ng/mL)	4.097 ± 4.409	5.348 ± 6.151	0.245
Troponin (24 h) (ng/mL)	3.711 ± 5.091	4.087 ± 4.822	0.433
C-reactive protein (before CPB) (mg/L)	10.75 ± 14.65	6.50 ± 6.30	0.911
C-reactive protein (on CPB) (mg/L)	6.82 ± 9.62	4.84 ± 4.38	0.871
C-reactive protein (6 h) (mg/L)	16.59 ± 18.30	22.30 ± 15.44	0.055
C-reactive protein (12 h) (mg/L)	67.27 ± 25.06	70.95 ± 25.34	0.527
C-reactive protein (24 h) (mg/L)	160.95 ± 35.93	170.30 ± 45.97	0.291
Lactate Dehydrogenase (before CPB) (IU/L)	142.10 ± 23.52	143.85 ± 31.36	0.843
Lactate Dehydrogenase (on CPB) (IU/L)	266.12 ± 112.47	156.45 ± 25.54	0.001
Lactate Dehydrogenase (6 h) (IU/L)	406.75 ± 178.54	287.20 ± 81.02	0.010
Lactate Dehydrogenase (12 h) (IU/L)	355.95 ± 89.07	263.70 ± 72.86	0.001
Lactate Dehydrogenase (24 h) (IU/L)	316.50 ± 112.51	254.25 ± 74.55	0.046

Note: Values are presented as mean ± standard deviation (SD). CPB, Cardiopulmonary Bypass, CPB; MiECC, Minimal Invasive Extracorporeal circulation.

Troponin levels at 6 h post-CPB were also significantly lower in the HS-group (3.188 ± 2.684 ng/mL vs. 4.645 ± 3.422 ng/mL, *p* = 0.038). Other parameters, such as creatinine and C-reactive protein, showed no significant differences at most time points (Table 6). Mechanical ventilation duration, ICU stay, and hospital stay was similar in the two groups (Table 7).

**Table 7:** Mechanical ventilation and length of stay in ICU comparison.

	Hybrid system (*n* = 20)	MiECC (*n* = 20)	*P* value
Mechanical ventilation (hours)	11.9 ± 3.79	12.5 ± 2.41	0.555
Length of stay in ICU (days)	1.7 ± 1.17	1.9 ± 1.16	0.592
Hospital stay (days)	6.5 ± 1.84	6.5 ± 2.09	1.000

MiECC, Minimal Invasive Extracorporeal circulation; ICU, Intensive Care Unit.

## Discussion

This study compares the HS and MiECC systems in patients undergoing isolated CABG. With matched demographic and clinical characteristics across groups, including age, gender distribution, body surface area, and left ventricular ejection fraction any observed differences can more confidently be attributed to the performance of the HS versus MiECC in the perioperative setting; in a sense, HS is no inferior to MiECC.

A primary outcome was the CPB time, which, though not reaching statistical significance (*p* = 0.134), was shorter in the HS-group compared to the MiECC group (93.35 ± 33.06 min vs. 108.65 ± 30.02 min). Shorter CPB durations are clinically advantageous, as prolonged CPB times are associated with higher risks of inflammation and potential complications [3, 4]. In this context, the HS may offer a time-efficient alternative to the MiECC technique without compromising the safety and quality of care. Both groups requiring a similar number of anastomoses and exhibited comparable hemoglobin levels at all-time points-preoperative, perioperative, and postoperative (at 6, 12 and 24 h) – suggesting that oxygen carrying capacity was similarly managed.

No significant difference in transfusion requirements was observed between the HS and the MiECC. This transfusion strategy likely reflects specific institutional practices and patient profiles, which may differ from those of other centers. In our institution, the transfusion trigger generally aligns with the nadir hemoglobin level, which is influenced by patient-specific factors such as age, comorbidities, and clinical status [7]. According to current literature, transfusion triggers in cardiac surgery often range from haemoglobin levels of 7–8 g/dL, depending on the patient’s hemodynamic stability and risk profile [3, 5]. These variations in transfusion practices and patient selection criteria across institutions can significantly impact transfusion rates, as also observed in multi-center registries. In terms of oxygenation, the HS achieved an indexed oxygen delivery (DO_2_i) of 311.60 ± 28.29 mL/min/m^2^, which, while not statistically superior to the MiECC group (332.25 ± 57.04 mL/min/m^2^, *p* = 0.275), highlights the HS’s capability to maintain stable oxygenation. Notably, the PaO_2_ was higher in the MiECC-group, because HS-group used PaO_2_ monitoring control with Quantum Ventilation Module by Spectrum medical to prevent the deleterious effects of hyperoxia (210.90 ± 49.64 mmHg vs. 177.70 ± 70.41 mmHg, *p* = 0.093).

Hyperoxia refers to a state where oxygen levels in the blood or tissues are excessively high. It is generally defined by a PaO₂ above the normal physiological range, typically greater than 100 mmHg. Hyperoxia often occurs when patients receive supplemental oxygen, especially at high concentrations or over prolonged periods, such as during surgery (including CABG with CPB), mechanical ventilation, or emergency care [2]. In clinical practice, the focus is often on balancing oxygen delivery to avoid hypoxia (low oxygen) while preventing hyperoxia. Excessive oxygen can lead to cellular and tissue damage due to increased production of reactive oxygen species (ROS), contributing to oxidative stress, inflammation, and other complications in both acute and long-term settings [1, 4, 5].

The HS-group employed hypobaric oxygenation during CPB, aiming to reduce gaseous micro-emboli formation and minimize stress on blood components and the endothelium, while maintaining effective oxygenation, hypobaric oxygenation is achieved by maintaining an oxygen pressure lower than standard levels in the oxygenator [8].

Biochemical parameters further illustrate differences in tissue response between the two systems. Lactate levels at the 6th hour after CPB was lower in the HS-group (2.85 ± 1.20 mmol/L vs. 4.04 ± 1.40 mmol/L, *p* = 0.009), indicating tissue metabolic stability. Similarly, troponin levels indicative of myocardial injury was significantly lower in the HS group at the 6th hours after CPB (3.188 ± 2.684 ng/mL vs. 4.645 ± 3.422 ng/mL, *p* = 0.038), suggesting reduced myocardial stress. This difference could be attributed to several specific features of the HS. One potential mechanism is the system’s stable oxygenation capability. The HS maintains a consistent oxygen delivery due to its dual-chamber design and collapsible soft bag reservoir, which allows for more precise control of oxygenation and blood volume during CPB. This stability reduces fluctuations in tissue perfusion and oxygen supply, which are known contributors to myocardial ischemia and injury, especially in high-risk patients undergoing cardiac surgery.

Additionally, the minimized blood-air interface in the HS further contributes to lower troponin levels by limiting contact between the blood and external surfaces, which helps reduce systemic inflammatory responses. In traditional CPB setups, exposure to foreign surfaces and air can lead to the activation of inflammatory pathways, ultimately increasing oxidative stress and myocardial workload. By minimizing this exposure, the HS likely reduces myocardial oxygen demand and preserves cardiac tissue integrity, resulting in lower troponin release postoperatively. Together, these factors suggest that the HS’s design supports myocardial protection through enhanced oxygenation stability and reduced inflammatory activation [8, 9]. Future studies focusing on myocardial injury markers in larger cohorts could further validate these findings and clarify the protective mechanisms inherent in the HS.

However, an interesting contrast was observed in LDH levels and trends, which were lower in the MiECC group during and after CPB compared to the HS group. LDH serves as a marker of cellular damage, and elevated levels may indicate cell stress or injury [10]. The lower LDH levels in the MiECC group suggest that this system may better preserve cellular integrity, potentially due to its established mechanisms to mitigate blood-air interface exposure and microemboli formation. This finding warrants further exploration, as it may reveal additional nuances regarding cellular preservation between the two systems. Additional parameters, including mean arterial pressure, creatinine, and C-reactive protein showed no significant differences between groups, indicating stable hemodynamic and inflammatory responses. ICU and hospital length of stay, along with mechanical ventilation durations, were also similar, reflecting comparable recovery time between the two systems.

A critical difference between the systems lies in the management of venous drainage. The MiECC system, being a closed circuit, often requires additional time to stabilize venous drainage prior to aortic cross-clamping. This stabilization is influenced by factors such as patient positioning, venous cannula placement, and adjustments to the active drainage system connected. These adjustments can introduce a delay, especially when ensuring proper venous return to maintain circuit integrity and avoid circuit air entrapment. In contrast, the HS system, simplifies venous drainage vision and management. The system allows for more flexible positioning and quicker adaptation to changes during the procedure, reducing the need for additional stabilization time. This efficiency in setup and operation could translate into shorter CPB times, even if this was not significant in the current analysis. Further multicentre studies with larger samples are recommended to validate these findings and to assess long-term outcomes of each system in broader patient populations.

## Conclusion

The HS demonstrated comparable, if not advantageous, results when matched against the MiECC in patients undergoing isolated CABG. Indexed oxygen delivery was similar in the two groups, while differences in partial pressure of oxygen and overall ICU and hospital stay durations were minimal between groups. Additionally, the HS offers a more intuitive management approach compared to the MiECC technique, potentially simplifying perioperative handling and reducing the learning curve for clinical teams. These results underscore that the HS is not inferior to MiECC; on the contrary, it presents an efficient and user-friendly alternative with promising outcomes. Further studies with larger sample sizes are recommended to confirm these findings and to assess potential advantages in broader clinical settings.

## Data Availability

The data presented in this study are available upon request from the corresponding author due to ethical considerations.
